# Pre‐Obesity and Preclinical Obesity: Disease‐Specific Concepts or Generalisable Frameworks for Chronic Disease Classification?

**DOI:** 10.1002/dmrr.70238

**Published:** 2026-10-22

**Authors:** Matteo Monami, Francesco Giorgino, Claudio Borghi, Benedetta Ragghianti, Valeria Guglielmi, Paolo Sbraccia

**Affiliations:** ^1^ Diabetology Unit Azienda Ospedaliero‐Universitaria Careggi Florence Italy; ^2^ Department of Precision and Regenerative Medicine and Ionian Area Section of Internal Medicine Endocrinology Andrology and Metabolic Diseases University of Bari Aldo Moro Bari Italy; ^3^ Medical and Surgical Sciences Deparment University of Bologna‐IRCCS AOU S. Orsola Bologna Italy; ^4^ Department of Systems Medicine University of Rome Tor Vergata Rome Italy; ^5^ Internal Medicine Unit and Obesity Center University Hospital Policlinico Tor Vergata Rome Italy

**Keywords:** chronic disease, disease classification, obesity, type 2 diabetes

The Lancet Commission on Clinical Obesity proposes a distinction between conditions that primarily represent future health risk and those that constitute established illness. Its separation of pre‐obesity, preclinical obesity and clinical obesity provides a useful framework for discussing the relationship between adiposity, disease definition and disease staging [1].

At a conceptual level, the Commission describes three states. Pre‐obesity refers to a condition associated with increased future risk but not yet meeting criteria for obesity. Preclinical obesity refers to individuals who already meet obesity criteria but have not developed obesity‐related organ dysfunction, clinical manifestations or limitations in activities of daily living. Clinical obesity is defined by the presence of such consequences [1].

Although this framework was developed for obesity, its underlying logic is not unique. Many chronic diseases can be viewed as progressing through a risk stage, a biologically defined disease stage without clinically evident consequences and a stage characterised by organ dysfunction and/or complications. Importantly, using diabetes and hypertension as comparators does not imply biological or nosological equivalence with obesity. Rather, these conditions provide a conceptual benchmark against which the strengths and limitations of the proposed distinction between risk, preclinical disease and clinical disease can be examined.

## 1 A General Three‐Stage Model

Current classifications of diabetes and hypertension already recognise risk states that precede disease. Prediabetes identifies individuals with glycaemic values above the normal range but below accepted diagnostic thresholds for diabetes mellitus (HbA1c 5.7%–6.4% [42–47 mmol/mol]) [2]. Similarly, elevated (or high‐normal) blood pressure (120–139/70–89 mmHg) identifies individuals at increased cardiovascular risk who do not yet meet diagnostic criteria for hypertension [3].

If the Commission's framework were applied consistently to these conditions, an intermediate category would emerge after diagnostic thresholds are crossed but before complications develop. Individuals with diabetes who have no retinopathy, nephropathy, neuropathy or functional impairment could be described as having preclinical diabetes. Likewise, individuals with hypertension but without hypertension‐mediated organ damage could be considered to have preclinical hypertension.

Under such a model, disease progression would involve three stages: a risk state (prediabetes or prehypertension), a biologically defined disease state without clinically manifest consequences (preclinical diabetes or preclinical hypertension) and a disease state with established complications (clinical diabetes or clinical hypertension). In this sense, the distinction between preclinical and clinical disease is potentially generalisable rather than obesity‐specific. However, such conceptual generalisability should not be interpreted as evidence that obesity, diabetes and hypertension are biologically equivalent disease entities.

## 2 Is Obesity Different From Diabetes and Hypertension?

The broader applicability of this framework highlights an important difference between obesity and conditions such as diabetes and hypertension.

In diabetes, diagnostic thresholds are anchored to biological markers with a well‐established relationship to disease‐specific complications. Hyperglycaemia is measured directly, and diagnostic cut‐offs are supported by evidence linking glycaemic levels to diabetes‐specific outcomes such as diabetic retinopathy [2]. Similarly, hypertension is defined by blood pressure measurements that predict future cardiovascular and renal events [3]. In both cases, disease definition precedes disease staging even though it is informed by it: accepted biological or biochemical criteria identify the disease, whereas complications truly determine its severity and prognosis, and ultimately staging.

Obesity lacks comparable anchors. No universally accepted biological marker equivalent to HbA1c or blood pressure exists. BMI, waist circumference, waist‐to‐height ratio, body fat percentage, visceral adipose tissue and ectopic fat accumulation each capture different dimensions of adiposity, yet none has achieved universal acceptance as the measure defining obesity as a disease [1, 4, 5, 6].

Furthermore, obesity lacks a characteristic disease‐specific complication analogous to diabetic retinopathy or hypertension‐mediated organ damage (e.g., left ventricular hypertrophy). Instead, obesity‐related complications encompass a heterogeneous and ample spectrum of conditions including type 2 diabetes, MASLD, obstructive sleep apnoea syndrome, osteoarthritis, heart failure, infertility and several malignancies [1].

Importantly, this heterogeneity is not limited to complication frequency but extends to complication type. Individuals with similar levels of adiposity may develop entirely different clinical manifestations. A person with severe obesity may predominantly experience mechanical complications such as osteoarthritis or obstructive sleep apnoea syndrome while remaining free of diabetes or cardiovascular disease. Conversely, an individual with only modest elevations in BMI but substantial central adiposity may develop type 2 diabetes, MASLD, or cardiovascular disease despite the absence of major mechanical complications. Moreover, progression from excess adiposity to clinically relevant dysfunction is neither uniform nor inevitable. Individuals with comparable degrees of adiposity may differ substantially in their long‐term trajectories because of differences in fat distribution, adipose tissue biology, age, sex, genetic susceptibility, physical fitness, metabolic status and other individual or contextual factors. Thus, unlike diabetes and hypertension, increasing adiposity and BMI do not necessarily lead to a coherent spectrum of disease‐specific outcomes. This observation raises a fundamental question. If obesity lacks both a universally accepted biological marker and a characteristic complication anchored to diagnostic thresholds, should obesity‐related complications define the disease itself, or should they primarily define its stage and severity? The Commission partially adopts the former position by defining ‘clinical’ obesity through the presence of obesity‐related organ dysfunction, clinical manifestations or limitations in daily living. Consequently, complications contribute not only to staging but also to determining whether clinically significant disease exists. By contrast, in most chronic diseases, complications modify stage and prognosis but do not determine the existence of disease. A patient with newly diagnosed diabetes remains diabetic despite the absence of retinopathy, just as a patient with hypertension remains hypertensive despite the absence of target‐organ damage. The challenge may lie not only in distinguishing obesity into preclinical and clinical stages, but also in accurately defining the underlying disease entity before any staging framework is applied.

Excess adiposity without demonstrable dysfunction may, at least in some individuals, represent susceptibility to future disease rather than disease itself. Nonetheless, the identification of increased risk may still justify intervention to prevent complications. Disease definition and the indication for prevention should therefore be considered related but distinct questions.

## 3 Does Early Treatment Matter in Pre‐Disease State and Preclinical Disease?

### 3.1 Prediabetes and Diabetes

Preventive pharmacological treatment may be considered in selected individuals with prediabetes who are judged to be at particularly high risk of progression to overt diabetes, including those with HbA1c levels approaching the diagnostic threshold (e.g., ≥ 6.0%), with multiple glycaemic abnormalities (impaired fasting glucose and impaired glucose tolerance), especially when lifestyle intervention is insufficient [2].

However, once diagnostic glycaemic thresholds are exceeded, management is prompted to restore glycaemic levels possibly below the diagnostic threshold itself, whereas the presence of complications informs on prognosis and guides treatment intensity [2]. The UK Prospective Diabetes Study (UKPDS) enrolled patients with newly diagnosed type 2 diabetes, most of whom were not characterised by clinically apparent microvascular or macrovascular complications at baseline [7]. An intensive glycaemic management reduced microvascular complications and generated long‐term reductions in myocardial infarction and all‐cause mortality during post‐trial follow‐up, a phenomenon later described as the legacy effect [7, 8, 9].

### 3.2 Prehypertension (i.e., Elevated Blood Pressure) and Hypertension

Similarly, in individuals with blood pressure levels above the optimal range but below the diagnostic threshold for hypertension, following ESC guidelines, treatment may or may not be warranted depending on overall cardiovascular risk. However, once hypertension is diagnosed, target‐organ damage influences prognosis and treatment intensity but does not generally determine whether the disease is present or deserves anti‐hypertensive treatments [3]. In fact, randomised trials and meta‐analyses in hypertension, including SPRINT and subsequent collaborative analyses, demonstrated that blood pressure lowering reduces cardiovascular events, heart failure and mortality before many traditional complications have developed [10, 11, 12]. Treatment is initiated precisely because future disease burden can be prevented.

These examples illustrate a general principle: intervention is often most effective before complications emerge. Whether obesity should be approached differently remains uncertain. The key question is therefore whether in obesity the absence of dysfunction identifies a benign state or an opportunity for prevention. Although definitive obesity‐specific prevention trials analogous to UKPDS [7] and SPRINT [10] are lacking, the SELECT trial [13] demonstrated that targeting obesity can reduce major cardiovascular events, reinforcing the concept that excess adiposity is not merely a risk marker but a modifiable disease process. Admittedly, SELECT [13] was conducted in individuals with established cardiovascular disease, and the boundary between primary and secondary prevention remains open to debate.

Nonetheless, these findings raise important questions about approaches that place excessive emphasis on waiting for clinically overt dysfunction before recognising obesity as a disease state. Resolving this issue requires a clearer understanding of which adiposity‐related phenotypes predict future disease and which do not.

## 4 Conclusion

The distinction between pre‐obesity, preclinical obesity and clinical obesity offers a useful framework for recognising the heterogeneity of adiposity‐related conditions. More broadly, it reflects a generalisable model in which chronic diseases can be conceptualised as progressing from a risk state to a biologically defined disease state and ultimately to a stage characterised by organ dysfunction and/or complications.

However, applying this framework across diseases highlights a challenge that may be unique to obesity. Unlike diabetes and hypertension, obesity lacks universally accepted biological markers, characteristic disease‐specific complications, and diagnostic thresholds anchored to clearly defined outcomes. In addition, individuals with similar levels of adiposity may develop markedly different complications, reflecting differences in fat distribution, adipose tissue biology and individual susceptibility.

The debate surrounding preclinical obesity should therefore focus more on disease definition itself. Before obesity can be confidently subdivided into preclinical and clinical stages, a more fundamental question may need to be addressed: what constitutes obesity?

## Author Contributions

M.M. and P.S. conceived the manuscript, developed its central arguments, coordinated the project and prepared the first draft. F.G., C.B., and V.G. critically revised the intellectual content of the manuscript and contributed to the interpretation of the evidence and refinement of the arguments. All authors reviewed and approved the final version of the manuscript and accept responsibility for its content.

## Conflicts of Interest

The authors declare the following competing interests: M.M. has served on advisory boards for AstraZeneca, Boehringer Ingelheim, Eli Lilly and Novo Nordisk. He has received honoraria for lectures, presentations or educational activities from AstraZeneca, Boehringer Ingelheim, Eli Lilly, Viking and Novo Nordisk. F.G. has served on advisory boards for AstraZeneca, Biomea, Boehringer Ingelheim, Eli Lilly, Kayothera, Lifescan, Merck Sharp & Dohme, Medtronic, Novo Nordisk, Roche Diabetes Care and Sanofi; as consultant for Eli Lilly, Novo Nordisk and Kayothera, he has received honoraria for lectures, presentations or educational activities from AstraZeneca, Boehringer Ingelheim, Eli Lilly, Lifescan, Merck Sharp & Dohme, Medtronic, Novo Nordisk, Roche Diabetes Care and Sanofi. He has received research support from Eli Lilly and Roche Diabetes Care and support for medical writing or statistical analysis from Eli Lilly, Novo Nordisk, Roche Diabetes Care and Sanofi. He also serves on the boards of the European Association for the Study of Diabetes (EASD)/European Foundation for the Study of Diabetes (EFSD) and the European Diabetes Forum (EUDF). C.B. has received consulting fees from Novartis Pharma, Alfasigma, Grünenthal, Menarini Corporate and Laboratoires Servier, and lecture fees from Laboratoires Servier, Takeda, Astellas, Teijin, Novartis Pharma, Berlin‐Chemie and Sanofi. V.G. has received consulting fees, speaker honoraria and advisory board fees from Novo Nordisk and Eli Lilly, and travel support from Chiesi. P.S. has received honoraria and consulting fees from Boehringer Ingelheim, Chiesi, Novo Nordisk, Eli Lilly, Pfizer and Roche for participation in advisory boards.

## Data Availability

The authors have nothing to report.
